# How to navigate fieldwork

**DOI:** 10.7554/eLife.94879

**Published:** 2023-12-05

**Authors:** Hella Péter

**Affiliations:** 1 School of Anthropology and Conservation, University of Kent Canterbury United Kingdom

**Keywords:** sparks of change, being neurodivergent in academia, neurodiversity, fieldwork, ADHD, medication

## Abstract

A PhD student recounts what she has learned from managing her ADHD between the office and the rainforest.

It’s 11:58am in a Ugandan rainforest, and the morning chill has finally given way to a dry season heat that wraps around me like a blanket. The chimpanzees we’re observing are lounging on the ground, napping, grooming, some of the young ones playing. Up in the trees, a bird is whistling its distinctive four-note song. A small snail crawls on a mossy root in front of me, its ornate shell a bright warning of vibrant reds, blacks and yellows.

And I’m about to touch that snail.

My wristwatch beeps noon, snapping me out of my gastropod-bothering trance. The morning dose of my ADHD medication has worn off by now, as I took it around 7am when we first started looking for the chimpanzees. During fieldwork, they’re the ones who dictate when I take my medication; I need to be at my best while collecting data, so I don’t miss a detail of their behavior – or touch suspiciously colorful wildlife during occasional lulls in activity. I reach into my bag and find the blister pack stashed in the small zip pocket. Thumbing over it, I count five pills left. I’ll be working in the forest for the next four days, so I need to remember to add in another pack.

Back at my desk at the University of Kent in the UK, my medication does what most people would expect: it helps me focus, remember things and resist climbing office furniture. When I’m in the field, however, my hyperactivity and boredom are less of a problem. Sure, I may sometimes need to sit quietly for hours when around chimpanzees less habituated to humans, but overall, we easily cover 10 km a day to observe animals whose social life can rival a soap opera. In fact, my brain welcomes the easy, daily to-do list of fieldwork, the rigid routine and the abundant green that quiets my racing thoughts and allows me to truly relax. In the forest, some of my other ADHD traits become an issue instead, such as my inattention, ‘wonky’ risk assessment ability and poor motor coordination skills.

I think back to my first field season five years ago, before I was officially diagnosed and on medication. I kept zoning out and stumbling over logs, chatting incessantly and sticking my hands into places I probably shouldn’t have (looking at you, ominous tree hole!). Struggling to keep track of the forest paths, I spent most of my time trailing like a lost duckling after Kizza, the field assistant I work with. Now, I can easily notice the landscape details that will come together in my head like an illustrated map, and I’m much less accident-prone overall. In the forest, I need my medication for my safety, not just my work.

Still, bringing my precious blister packs with me hadn’t been easy. While ADHD meds are safe to take when prescribed, some of them can be misused by people who do not have ADHD. As a result, many countries tightly regulate how many pills I can carry (an issue for long field trips), or even ban my medication altogether. On top of the regular pile of paperwork required before fieldwork, I needed to confirm whether I could do my research in Uganda without risking getting arrested for drug possession, as well as put together flight itineraries that excluded stopovers in countries where my treatment is illegal. With this information not easily accessible online, I resorted to poring over obscure travel forums and reaching out to the Ugandan consulate.

Then there was also the ‘people problem’. In the UK, I had learned how to carefully share my diagnosis with my co-workers, typically choosing to focus on common office-related symptoms like why I may sit weird and walk around, or why I may need quiet, uninterrupted time to work. All my go-to phrases would need adjusting for my colleagues in the field. Still, I drew some confidence from the fact that they had already seen me at my clumsiest and most forgetful three years before; in the end, explaining that it was because of a medical condition wasn’t too much of a new thing. What I found was that, while people in Uganda generally know less about neurodiversity than people in the UK, it meant that preconceptions and stereotypes were less common there too. Questions about my treatment typically came from people concerned about my health. In the UK, however, I’d observed a certain skepticism surrounding ADHD medication, particularly in academic settings where it’s often referred to as a ‘study drug’.

Back in the forest, I check my water bottle for drowned ants — all clear, thankfully — and swallow my afternoon dose with a lukewarm sip. Above us, a two-year-old chimpanzee called Teddy does a headstand peering down at me. Her mother is having her siesta, and clearly, she is bored. My meds will kick in by the time the heat lifts and the troop are on the move again, requiring all my focus. Until then, Teddy’s antics will keep me entertained.

In the end, fieldwork has taught me a lot about my neurodivergence. This has gone beyond learning how to manage my ADHD in the field (Box 1). Back in the UK, I’m trying to incorporate into my daily routine some of the elements that make my brain thrive in the forest, such as exercise and being in nature. More importantly, my trips have highlighted the fluidity of my ADHD, which I had tended to see as fixed before. They’ve shown me that a weakness in the office can be a strength in the field. I’ve realized that, as put forward by the social model of disability, my negative symptoms partly come from a clash between my environment and how my brain works, instead of some fundamental internal faults. That it can be what’s around me that needs changing, rather than what’s within.

Box 1. **Tips for managing ADHD and ADHD medication in the field.**

- Check rules and regulations concerning your medication in the country you are traveling to, and any countries you are traveling through.
- Keep medication in your hand luggage, in its original packaging, with an official letter from a doctor explaining what it is, how much there is, and why you need that amount.
- Think about the first few weeks in the field as a pilot study – a drastically different environment and daily routine will likely mean a different profile of primary symptoms to deal with. You might also have to change when you take your medication.
- Remember, how much you disclose, when and to whom, is entirely up to you.
- When disclosing to someone new, focusing on the symptoms you experience over medical jargon is typically more helpful (e.g.: ‘I have trouble remembering things and staying still’ instead of ‘I have executive function issues and deal with hyperactivity’), especially for people with little prior knowledge of ADHD.

Finally, I’ve learned that I’m not alone. After I started talking about my experiences on social media, many neurodivergent scientists reached out to share their story or ask for advice. Seeing how many of us are out there doing this work and excelling at it has given me hope that slowly but surely, the scientific community will recognize that we have our place in the field and become more accepting.

## About this article

This Sparks of Change article is part of a series of articles on being neurodivergent in academia, which includes a list of tips, resources and tools collated by neurodivergent scientists.

